# Palmitic acid inhibits vascular smooth muscle cell switch to synthetic phenotype via upregulation of miR-22 expression

**DOI:** 10.18632/aging.204334

**Published:** 2022-10-12

**Authors:** Yanchao Hu, Yajie Fan, Chunyan Zhang, Congxia Wang

**Affiliations:** 1 Department of Cardiovascular Medicine, The Second Affiliated Hospital of Xi'an Jiaotong University, Shaanxi, Xi'an 710004, China

**Keywords:** VSMC, palmitic acid, synthetic phenotype, miR-22

## Abstract

Synthetic phenotype switch of vascular smooth muscle cells (VSMCs) has been shown to play key roles in vascular diseases. Mounting evidence has shown that fatty acid metabolism is highly associated with vascular diseases. However, how fatty acids regulate VSMC phenotype is poorly understood. Hence, the effects of palmitic acid (PA) on VSMC phenotype were determined in this study. The effect of the PA on VSMCs was measured by live/dead and EdU assays, as well as flow cytometry. Migration ability of VSMCs was evaluated using transwell assay. The underlying targets of miR-22 were predicted using bioinformatics online tools, and confirmed by luciferase reporter assay. The RNA and protein expression of certain gene was detected by qRT-PCR or western blot. PA inhibited VSMC switch to synthetic phenotype, as manifested by inhibiting VSMC proliferation, migration, and synthesis. PA upregulated miR-22 in VSMCs, and miR-22 mimics exerted similar effects as PA treatment, inhibiting VSMC switch to synthetic phenotype. Inhibition of miR-22 using miR-22 inhibitor blocked the impacts of PA on VSMC phenotype modulation, suggesting that PA modulated VSMC phenotype through upregulation of miR-22 expression. We found that ecotropic virus integration site 1 protein homolog (EVI1) was the target of miR-22 in regulation of VSMC phenotype. Overexpression of miR-22 or/and PA treatment attenuated the inhibition of EVI1 on switch of VSMCs. These findings suggested that PA inhibits VSMC switch to synthetic phenotype through upregulation of miR-22 thereby inhibiting EVI1, and correcting the dysregulation of miR-22/EVI1 or PA metabolism is a potential treatment to vascular diseases.

## INTRODUCTION

Vascular function is largely dependent on vascular smooth muscle cells (VSMCs). Different from the skeletal muscle cells or cardiomyocytes, VSMCs remain possessing remarkable phenotypic plasticity in response to multiple stimuli [1]. VSMCs switch from a contractile state to a dedifferentiated, synthetic phenotype, playing crucial roles in several vascular diseases [2–4]. The synthetic phenotype induces migration to the intima and enhances proliferation and extracellular matrix protein synthesis, thereby resulting in an impaired contractility of VSMC [5]. Therefore, exploration of the underlying mechanisms involved in VSMC phenotypic switch regulation is important in vascular diseases.

There are multiple environmental stimuli have been identified as factors which lead to VSMC phenotype switch, such as growth factors, reactive oxidative species (ROS), and mechanical injury [6, 7]. Recent studies have shown that metabolites were also involved in regulation of VSMC phenotype [8]. For example, lactate, a product of glucose metabolism, was found to promote the synthetic phenotype of VSMCs, which links glucose metabolism to VSMC phenotypic switch [8]. Mounting evidence has shown that fatty acid metabolism is abnormal in vascular diseases, which plays an important role in the development of atherosclerosis and other vascular diseases [9, 10]. These advances suggest that fatty acids metabolism may play a role in regulation of VSMC phenotype. However, how fatty acids regulate VSMC phenotype is poorly understood. As the most common saturated fatty acid found in organism, palmitic acid (PA) serves as an energy source or component of partially biochemicals and cellular structures. The circulating level of PA is increased in metabolic disorders and correlated with the adverse outcomes of cardiovascular diseases [11–13]. Here, we aimed to examine the impacts and underlying mechanism of PA on VSMC phenotype.

VSMC phenotype switch has been widely studied in transcriptional and epigenetic levels [14, 15]. We were very interested in the growing evidence supporting a critical role for miRNAs in regulating VSMC differentiation and phenotypic switch [16, 17]. A series of miRNAs have been reported as regulators of VSMC phenotype, including miR-21 [18], miR-22 [16], miR-23b [19], miR-100 [20], miR-124 [16], miR-133 [21], miR-143/145 [22], miR-146a [23], miR-195 [24], miR-221/222 [25] and miR-424 [26]. Here, we found that PA inhibits VSMC switch to synthetic phenotype via upregulation of miR-22. These results suggested that PA plays a role in regulation of VSMC phenotype.

## METHODS

### Cell culture

Primary VSMCs were isolated from 8–10 weeks old male SD rat (weighed 170–250 g) thoracic aorta as reported previously [27]. Briefly, thoracic aortas were excised followed by phosphate buffered saline (PBS) washing for 3 times. After these, the aortic media layer was dissected, cut into pieces, and seeded onto a 6-well plate. Cells were maintained in DMEM supplemented with 10% fetal bovine serum (FBS), 1% penicillin and streptomycin at 37°C in a humidified incubator with 5% CO_2_ in atmosphere for 2 weeks. All animal procedures in this study were conducted in accordance with the National Institutes of Health Guidelines on the Use of Laboratory Animals, and were approved by the Xi’an Jiaotong University Second Affiliated Hospital.

### Live/dead cell assay

The 2-color fluorescence with the LIVE/DEAD Viability/Cytotoxicity kit (Molecular Probes) was used to quantify the living and dead cells in this study as directed by the manufacturer’s protocol. Briefly, cells were harvested after treatment, washed with PBS twice, and incubated with 300 μl of live/dead solution for half an hour at 37°C in the dark room. Then, the fluorescence was read using a microplate reader (FLUOstar^®^ Omega).

### EdU assay

Proliferation of VSMCs was analyzed using the Click-it EdU kit (C10086, Invitrogen, USA). Briefly, cells were seeded on the slides at a density of 1.0 × 10^3^ cells in 12-well plate each well. After treatment, cells were incubated with 50 μmol/L EdU solution at 37°C for 2 h. Then, cells were washed with cooled PBS for twice and fix 4% PFA at 4°C for 15 min. Following this, 100 μl Apollo reaction cocktail was added into cells followed by nucleus staining with Hoechst 33342 according to the manufacturer’s protocol. The fluorescence signal was then analyzed under a fluorescence microscope. EdU incorporation (%) = EdU positive cells/(EdU-positive cells + Hoechst-positive cells) ×100%.

### Apoptosis determination

The apoptosis of VSMCs was detected using an Annexin V-FITC apoptosis detection kit (C1062, Beyotime, China). Briefly, cells were collected after treatment, washed with cooled PBS twice, resuspended with 1 mL AnnexinV-FITC, and maintained for 10 min at room temperature according to the kit’s protocol. Following this, cells were subjected to flow cytometry analysis.

### Transwell assay

VSMCs were seeded in the upper chamber of transwell (12 μm) and placed in a 24-well plate at a density of 1.0 × 10^5^ cells/well in 200 μl DMEM contained with 0.5% FBS. The lower chamber was filled up with 600 μl DMEM contained with 10% FBS. After incubation with for 24 h, medium was discarded and the lower chamber membrane was fixed with methanol at room temperature for 15 min. Subsequently, cells were stained with 0.1 crystal violet-methanol solution for 15 min at room temperature. Finally, the migrated cells were pictured and calculated under a light microscope.

### Real-time reverse transcription PCR

RNAiso Plus reagent (Code No.: 9108, Takara) was used for the RNA isolation as the manufacturer recommended. The cDNA was synthesized using the isolated RNA (500 ng/sample) and amplification of certain genes was performed using a SYBR Green PCR kit (Takara) in a CFX200 (Bio-Rad) with the cycles of 95°C for 10 min and 40 cycles of 95°C for 5 s, 58°C for 30 s, and 72°C for 10 s. The mRNA level of each gene was normalized to housekeeping gene, namely, GAPDH or U6. The primer sequences are listed in Supplementary Table 1.

### Cell transfection

MiR-22 mimics (5′-AAGCUGCCAGUUGAAGAACUGU-3′), miR-23b mimics (5′-AUCACAUUGCCAGGGAUUACCAC-3′), miR-125b mimics (5′-UCCCUGAGACCCUAACUUGUGA-3′), negative control mimics (NC mimics, #miR1N0000001-1-10), miR-22 inhibitors (5′-ACAGUUCUUCAACUGGCAGCUU-3′), and NC inhibitors (#miR2N0000001-1-10) were synthesized by RIBOBIO Co., Ltd Chin (Guangzhou, China). Empty vector (pcDNA3.1) and EVI1 overexpression plasmid (pcDNA3.1-EVI1 OE) were purchased from GeneChem (Shanghai, China). miRNA mimics (100 nmol/L), inhibitors (200 nmol/L), or NC (5′-UUCUCCGAACGUGUCACGUTT-3′) (100 nmol/L) were transfected using Lipofectamine™ 3000 (Invitrogen) according to manufacturer’s instruction. After 60 h post-transfection, the transfected cells were harvested and utilized for further analyses.

### Dual-luciferase reporter assay

Wt and Mt ecotropic virus integration site 1 protein homolog (*EVI1*) 3′UTR sequence was acquired using PCRmethod, and then cloned into SpeI and HindIII sites of pMir-Report Luciferase vector (Applied Biosystems). The resulting construct was transfected (5ng) into 293T cells with 20 nM control mimics or miR-22 mimics using Lipofectamine-2000 (Invitrogen). After 24 h post-transfection, luciferase activity of cells was assessed using a Luciferase Assay System (Promega).

### Western blot

For immunoblotting, proteins were isolated from cells using RIPA buffer. Total protein extracts (15–50 μg) were separated using sodium dodecyl sulfate polyacrylamide gel electrophoresis and transferred onto polyvinylidene fluoride membrane. Membranes were then probed with anti-bax (1:2000; #ab32503; Abcam), bcl-2 (1:2000; #ab196495; Abcam), cleaved-caspase-3/caspase-3 (1:2000; #ab184787; Abcam), SM22α (1:2000; #ab14106; Abcam), calponin (1:500; #ab227661; Abcam), SMMHC (1:2000; #ab125884; Abcam), vimentin (1:2000; #ab92547; Abcam), collagen I (1:1000; #ab270993; Abcam), osteopontin (OPN; 1:1000; #ab63856; Abcam), LAMC1 (1:1000; #ab233389; Abcam), EVI1 (1:1000; #SAB2100723; Sigma), AKT3 (1:2000; #ab152157; Abcam), TP53INP1 (1:2000; #ab202026; Abcam), and β-actin (1:2000; #ab8226; Abcam) at room temperature for 1.5 h. Then, membranes were immersed with the HRP-conjugated secondary antibody at room temperature for 1 h. Following this, the BM chemiluminescence blotting system (Thermo Scientific) was used for detection and protein bands were quantified using Image J software (NIH, USA).

### Statistical analysis

All data are presented as mean ± standard deviation, and comparisons were performed using one-way ANOVA or two-way ANOVA followed by an unpaired *t*-test, as appropriate. *P* < 0.05 was considered statistically significant.

### Availability of data and materials

The datasets used and/or analyzed during the present study are available from the corresponding author upon reasonable request.

## RESULTS

### PA inhibited the synthetic phenotype in cultured VSMCs

**Figure 1:**
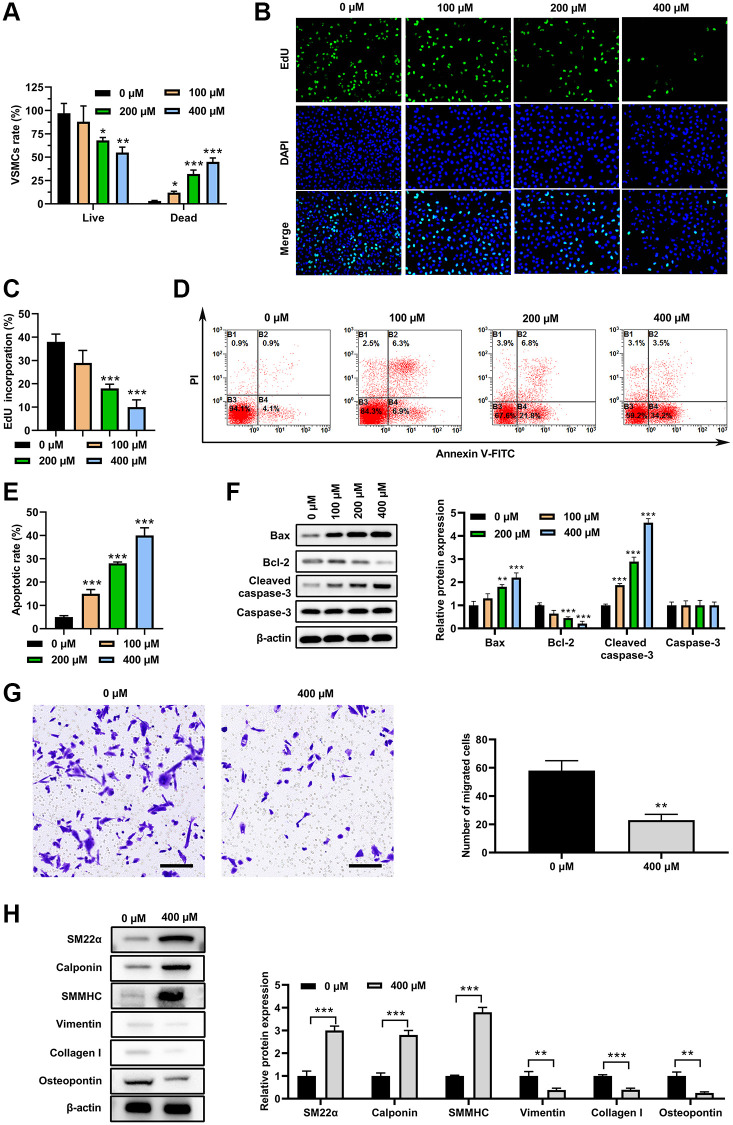
**PA inhibited VSMC switch to synthetic phenotype.** VSMC morphologies after PA treatments (0, 100, 200 or 400 μM) for 3d. (**A**) Live/dead cell assay of PA-treated VSMCs. (**B** and **C**). EdU assay to detect the proliferation on VSMCs treated with PA. (**D** and **E**). Flow cytometry to detect the apoptosis of PA-treated VSMCs. (**F**) Western blot determined the expression of apoptosis associated markers. (**G**) Transwell assay of PA-treated (200 μM) VSMCs. Scale bar = 100 μm. (**H**) Expression levels of SM22α, calponin, SMMHC, vimentin, collagen I, and osteopontin in PA-treated (200 μM for 3 d) VSMCs. *n* = 3. ^*^P < 0.05, ^**^P < 0.01.

VSMCs were treated with PA (0, 100, 200 or 400 μM) for 3 d. Live/Dead assay suggested that PA treatment decreased cell viability, and increased cell death in a dose-dependent manner in VSMCs (Figure 1A). Moreover, EdU assay suggested that PA treatment significantly decreased the EdU incorporation of VSMCs in a dose-dependent manner (Figure 1B and 1C). Further analysis indicated that PA treatment could significantly increase VSMCs apoptosis (Figure 1D and 1E). Western blot analysis presented that PA treatment markedly increase the Bax and cleaved-caspase-3 expression but decreased Bcl-2 expression (Figure 1F). These findings suggested that PA may inhibit the VSMC switch to synthetic phenotype. In addition, PA treatment (200 μM) suppressed the migration of VSMCs as detected by transwell assay (Figure 1G). Furthermore, PA treatment (200 μM) for 3 d increased protein levels for markers of the contractile phenotype, including α-SMA, calponin, and SMMHC, and decreased protein levels of the synthetic phenotype, including vimentin, collagen I, and osteopontin (OPN) (Figure 1H). These results reinforced the notion that PA inhibits the VSMC switch to synthetic phenotype.

### PA increased miR-22 expression in VSMCs

**Figure 2:**
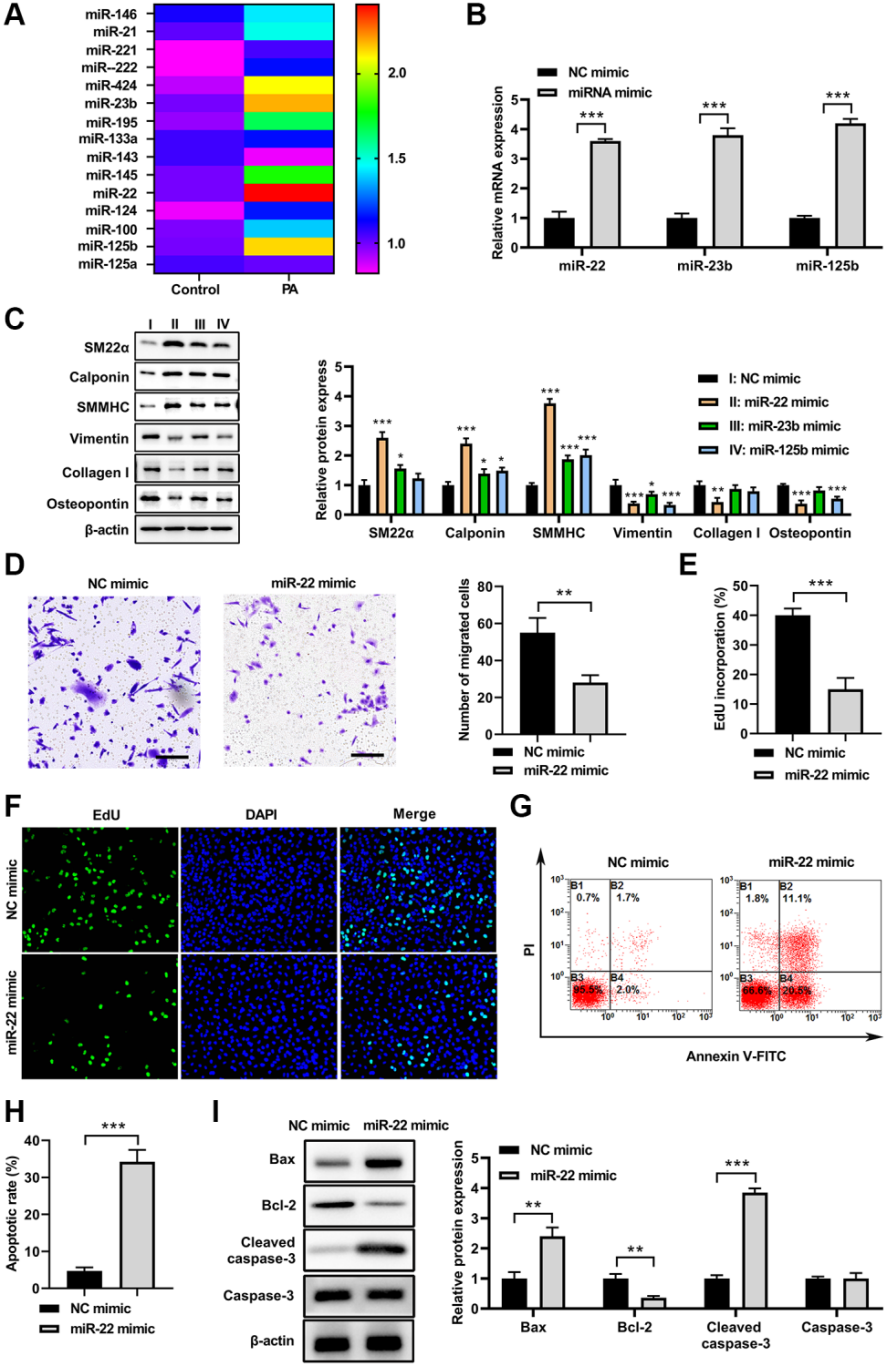
**PA increased miR-22 expression in VSMCs.** (**A**) MiRNAs levels in VSMCs treated with PA. (**B**) The mRNA levels of miR-22, miR-23b, and miR-125b in VSMCs after transfecting with their specific miRNA mimics. (**C**) The protein expression of SM22α, calponin, SMMHC, vimentin, collagen I, and osteopontin in VSMCs treated with miR-22, miR-23b or miR-125b mimics. (**D**) Migration ability of VSMCs transfecting with miR-122 mimics determined by transwell assay. Scale bar=100 μm. (**E**, **F**) The cell proliferation of VSMCs treated with miR-22 mimics detected by EdU assay. (**G**, **H**) Apoptosis of VSMCs treated with miR-22 mimics determined by flow cytometry. (**I**) The protein expression of bax, bcl-2, caspase-3, and cleaved-caspase-3 in VSMCs treated with miR-22 mimics. *n* = 3. ^*^P < 0.05, ^**^P < 0.01.

To test whether miRNA is involved in regulation of VSMC phenotype switch induced by PA, the reported miRNAs which are involved in alteration of VSMC phenotype switch were screened in PA-treated VSMCs. As shown in Figure 2A, 15 miRNAs were detected, and among these miRNAs, 3 miRNAs were increased and 1 miRNA were decreased in PA-treated VSMCs compared with that in untreated VSMCs. Following this, the top 3 increased miRNAs were overexpressed in VSMCs via using transfecting with their specific miRNA mimics, respectively (Figure 2B). Following this, the expression of synthetic and contractile markers was detected in VSMCs. As shown in Figure 2C, miR-22, miR-23b, and miR-125b mimics all increased the mRNA levels for SM22α, calponin, and SMMHC, and decreased mRNA levels of vimentin, collagen I, and OPN, suggesting that PA may inhibit the VSMC switch to synthetic phenotype via upregulation of these miRNAs. Specifically, miR-22 presented the most significant effect among these miRNAs. Thus, we had chosen miR-22 for the following investigation. Moreover, transwell assay suggested that increased expression of miR-22 mimics obviously inhibited the VSMCs migration (Figure 2D). Overexpression of miR-22 mimics also inhibited the proliferation of VSMCs (Figure 2E and 2F). In addition, flow cytometry suggested that overexpression of miR-22 mimic increased the apoptosis of VSMCs (Figure 2G and 2H). Correspondingly, the western blot analysis showed that miR-22 mimic increased the expression of Bax and clveaed-caspase-3, but decrease Bcl-2 expression (Figure 2I). These results suggested that PA may inhibit the VSMC switch to synthetic phenotype via upregulation of miR-22.

### miR-22 inhibitor abolished the effects of PA on VSMC phenotype switch

**Figure 3:**
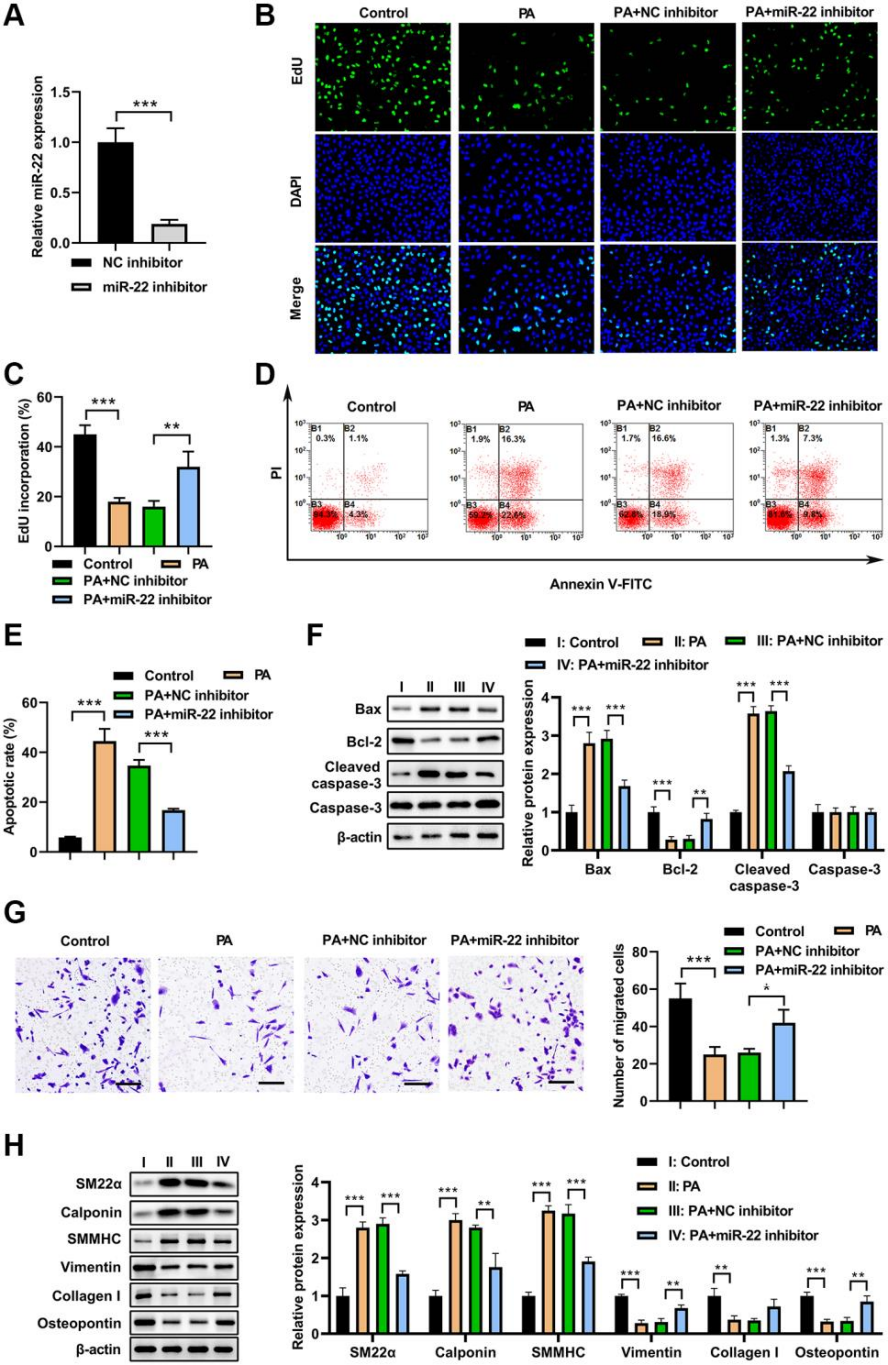
**miR-22 inhibitor abolished the effects of PA on VSMC phenotype switch.** (**A**) MiR-22 inhibitor decreased the miR-22 levels in VSMCs. (**B**, **C**) The proliferation of VSMCs transfected with miR-22 inhibitor or/and 200 μM PA. (**D**, **E**) The apoptosis of VSMCs transfected with miR-22 inhibitor or/and 200 μM PA. (**F**) The expression of apoptosis associated markers in VSMCs transfected with miR-22 inhibitor or/and 200 μM PA. (**G**) The migration of VSMCs transfected with miR-22 inhibitor or/and 200 μM PA determined by transwell assay. Scale bar=100 μm. (**H**) Western blot analyses showing that expression of SM22α, calponin, SMMHC, vimentin, collagen I, and osteopontin in PA-treated VSMCs transfected with miR-22 inhibitor. *n* = 3. ^*^P < 0.05, ^**^P < 0.01.

To test whether miR-22 is involved in the PA’s effects on VSMC phenotype switch, miR-22 inhibitor was used to inhibit the PA-upregulated miR-22. As shown in Figure 3A, miR-22 inhibitor decreased the miR-22 levels in VSMCs. As a result, PA treatment (200 μM) significantly inhibited the cell viability but increased apoptosis in VSMCs, while overexpression of miR-22 inhibitor attenuated the impacts of PA on the proliferation and apoptosis of VSMCs (Figure 3B–3E). Correspondingly, the western blot analysis presented that overexpression of miR-22 inhibitor attenuated the effect of PA in increasing bax and cleaved-caspase-3 expression, and decreasing bcl-2 expression (Figure 3F). Transwell analysis showed that overexpression of PA treatment significantly decreased the migration of VSMCs, but miR-22 inhibitor obviously aborted this enhancement (Figure 3G). In addition, the western blot analyses showed that PA treatment obviously accumulated the expression of SM22α, calponin, and SMMHC, but decreased the expression of vimentin, collagen I, and OPN; while overexpression of miR-22 inhibitor attenuated the effect of PA in VSMCs (Figure 3H). These results reinforced the notion that PA inhibits the VSMC switch to synthetic phenotype.

### EVI1 was the target of miR-22

**Figure 4:**
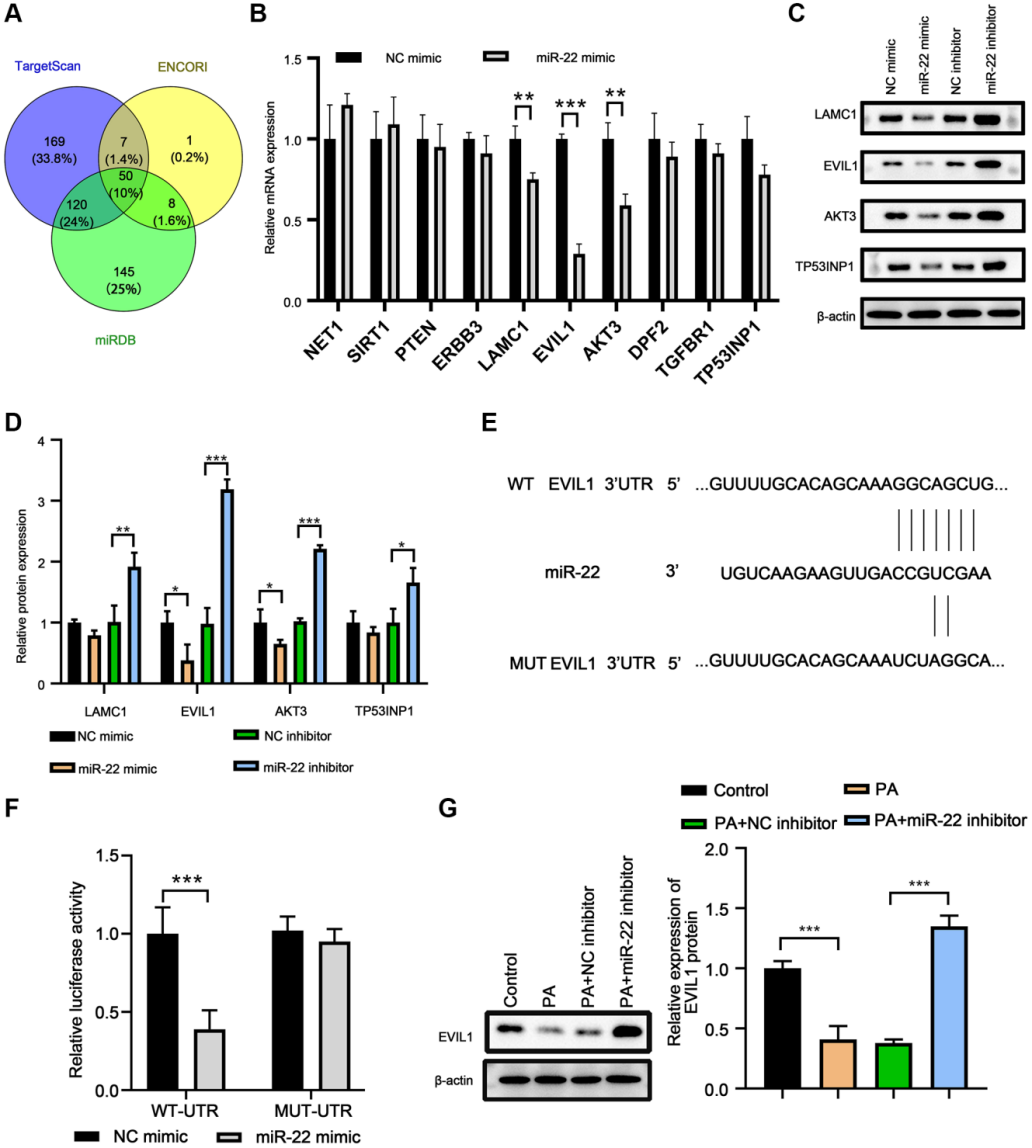
**EVI1 was the target of miR-22.** (**A**) Target genes of miR-22 were predicted by miRDB, TargetScan and ENCORI. (**B**) MRNA levels of target genes in VSMCs treated with miR-22 mimics. (**C**, **D**) Protein levels of SM22α, calponin, SMMHC, vimentin, collagen I, and osteopontin in VSMCs detected by western blot. (**E**) The complementary sequence between 3′-UTR of EVIL1 and miR-22. (**F**) Dual-luciferase reporter assay. (**G**) EVI1 protein level was detected by western blot in VSMCs treated with miR-22 inhibitor or PA. *n* = 3. ^*^P < 0.05, ^**^P < 0.01.

The potential target genes of miR-22 were predicted by miRDB, ENCOR1, and TargetScan. There were 50 candidates (Figure 4A), and 10 of them are associate with cell proliferation, migration, or apoptosis (Figure 4B). Among these candidates, overexpression of miR-22 reduced EVI1 mRNA levels significantly in VSMCs (Figure 4B). Western blot array showed the miR-22 mimic could significantly decrease the EVI1 expression, but miR-22 inhibitor largely enhanced the EVI1 expression (Figure 4C and 4D), indicating EVI1 acted as a candidate target of miR-22. This result was further confirmed by dual-luciferase reporter assay and presented that miR-22 reduced luciferase activity for EVI1 wild-type 3′UTR constructs but had no effect on the mutated binding site (Figure 4E and 4F). In addition, PA treatment abolished the EVI1 protein expression, while miR-22 inhibitor attenuated these downregulation, thereby upregulating EVI1 expression (Figure 4G). These results suggested that EVI1 is a target of miR-22.

### PA treatment enhanced the effect of miR-22 on abolishing EVI1mediated VSMC phenotype switch

**Figure 5:**
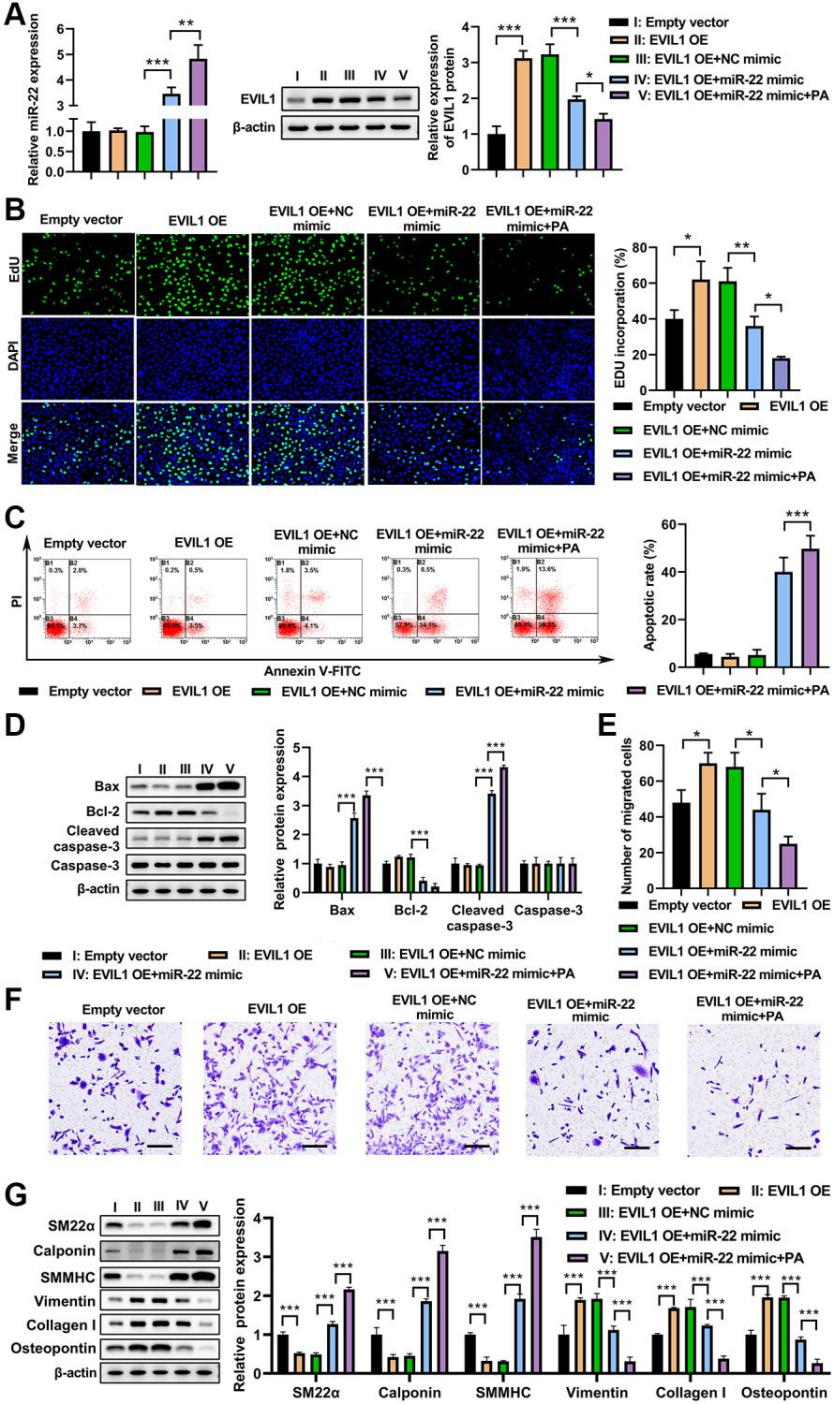
**PA treatment enhanced miR-22 mimic impacts on aborted the inhibitive effect of EVI1 on VSMC phenotype switch.** (**A**) Expression of miR-22 and EVIL1 in VMSCs treated with EVI1 or/and miR-22 overexpression followed by PA treatment. (**B**) Cell proliferation was detected by EdU in VSMCs with EVI1/miR-22 overexpression followed by PA treatment. (**C**) Flow cytometry to detect the apoptosis of VSMCs with EVI1/miR-22 overexpression followed by PA treatment. (**D**) Western blot to detect the apoptosis associated markers in VSMCs. (**E**, **F**). Cell migration was detected by transwell assay in EVI1/miR-22-overexpressed VSMCs followed by PA treatment. Scale bar = 100 μm. (**G**) Protein levels of SM22α, calponin, SMMHC, vimentin, collagen I, and osteopontin in VSMC in VSMCs with EVI1/miR-22 overexpression followed by PA treatment detected by western blot. *n* = 3. ^*^P < 0.05, ^**^P < 0.01.

To test whether EVI1 contributes to the effects of PA on VSMC phenotype switch, EVI1 and miR-22 were overexpressed in VMSCs followed by PA treatment. The qRT-PCR demonstrated that EVI1 overexpression did not change the levels of miR-22 in VSMCs, but PA treatment could enhance the upregulation of miR-22 (Figure 5A). Overexpression of miR-22 mimic significantly suppressed the EVI1 expression and PA treatment further enhanced this inhibition on the expression of EVI1 (Figure 5A). Moreover, PA treatment promoted cell proliferation as detected by EdU staining in VSMCs with EVI1 overexpression, while miR-22 mimic expression aborted this upregulation and PA treatment markedly enhanced this inhibitive effect mediated by miR-22 (Figure 5B). Flow cytometry analysis presented that overexpression of EVI1 had no obvious effect on the apoptosis of VSMCs, but miR-22 and PA treatment could enhance the apoptosis of EVI1 (Figure 5C). Similarly, EVI1 had no obviously effect on the expression of bax, bcl-2, caspase-3, and cleaved caspase-3, while miR-22 mimic and PA treatment could significantly promote the bax and cleaved-caspase-3 but decreased bcl-2 expression (Figure 5D). Transwell assays indicated that EVI1 significantly increased the migration of VSMCs, while miR-22 and PA treatment obviously attenuated this promotion to suppress the migration of VSMCs (Figure 5E and 5F). In addition, EVI1 also inhibited the contractile markers of SM22α, calponin, and SMMHC and promoted the synthetic markers of vimentin, collagen I, and OPN, while miR-22 and PA treatment attenuated these changes (Figure 5G). These results reinforced the notion that PA inhibits the VSMC switch to synthetic phenotype through regulation of miR-22/EVI1 axis.

## DISCUSSION

Mounting evidence has shown that disorder in fatty acid metabolism plays a casual role in the development of atherosclerosis and other vascular diseases [9, 10]. However, how fatty acid regulates VSMC phenotype switch has not been studied. Here, we found that PA, the most common saturated fatty acid in circulation, inhibited VSMC switch to synthetic phenotype, as manifested by inhibiting VSMC proliferation, migration, and synthesis. Mechanistically, PA inhibits VSMC switch to synthetic phenotype through upregulation of miR-22 by targeting EVI1. These findings suggested that PA plays a role in the regulation of VSMC phenotype, which may contribute to vascular health and diseases.

Several studies have shown that saturated fatty acids increase the risk of cardiovascular diseases [12, 28]. According to the previous study, the saturated fatty acids were usually regarded as a singular fatty acid group and they might have the same effects during the metabolism [29, 30]. However, some investigations focused on different biomarkers of risk of cardiovascular diseases found that not all SFAs exert the same effect, namely, studies do not seem to serve as a single role of PA in the development of cardiovascular diseases [31]. PA slightly elevated the LDL- and HDL-cholesterol, which is a significant predictor for cardiovascular disease [32, 33]. Although the role of PA in cardiovascular diseases needs to be further examined, these advances suggest that PA’s effects in cardiovascular health and disease cannot be easily identified as detrimental or beneficial. Here, we found that PA inhibited VSMC switch to synthetic phenotype, as manifested by inhibiting VSMC proliferation, migration, and synthesis, suggesting that PA may exert beneficial effects on vascular health and diseases, which should be identified by further studies.

Recent studies support a critical role of miRNAs in regulating VSMC differentiation and phenotype switch, and miR-22 is one of the miRNAs which inhibits VSMC switch to synthetic phenotype [16, 17]. miR-22 is previously demonstrated as a tumor suppressor, but later has been concerned as a prohypertrophic miRNA [34, 35]. A recent study documented that miR-22 playing key role in the regulation role in VSMC biological activity [36]. In addition, it has also been reported that miR-22 involved in VSMC phenotypic modulation, which induces VSMC contractile gene expression, but inhibits VSMC proliferation and migration [17]. These findings indicated that miR-22 serves a key role in regulation of cardiovascular function. Here, we show that PA increased miR-22 expression in VSMCs, and inhibition of miR-22 abolished the PA’s effects on modulation of VSMC phenotype. It has been reported that transforming growth factor-β1 (TGF-β1) transcriptionally modulates miR-22 expression in VSMCs via a P53-dependent mechanism [17]. Whether PA regulates miR-22 expression through TGF-β1 needed further investigation. Indeed, there is evidence that PA treatment increases TGF-β1 in other cells [37]. These findings suggested that PA modulate VSMC phenotype via upregulating miR-22, which serves a crucial role vascular function regulation.

Previous studies demonstrated that EVI1 functions as a transcriptional regulator to modulate several biological processes, including hematopoiesis, apoptosis, development, differentiation and proliferation [38, 39]. Here, we have found that EVI1 serves as a target gene of miR-22 to modulate VSMC phenotype switch. Further analysis showed that EVI1 transcriptionally inhibits VSMC-specific genes to modulate the VSMC phenotype switch, including SMαA, SM22α, SRF, and Myocd [17]. In addition, inhibiting EVI1 abolished the effects of miR-22 and PA in modulation of VSMC phenotype. These findings suggested that miR-22/EVI1 signaling axis plays a key role in VSMC phenotypic switch and correcting the dysregulation of miR-22/EVI1 or PA could be a potential treatment to vascular diseases.

## CONCLUSION

Taken together, we found that PA inhibits VSMC switch to synthetic phenotype through upregulation of miR-22 expression. In addition, miR22 inhibits VSMC switch to synthetic phenotype by targeting EVI1. These findings suggested that PA plays a role in regulation of VSMC phenotype, which may contribute to maintenance of vascular health and prevention of vascular diseases.

## Supplementary Materials

Supplementary Table 1
