# Supplementary material for: Palmitic acid inhibits vascular smooth muscle cell switch to synthetic phenotype via upregulation of miR-22 expression
Source: Aging (Albany NY). 2022 Oct 12;14(19):8046–60. doi: 10.18632/aging.204334 (PMC9596196; doi:10.18632/aging.204334)
Supplement: Supplementary Table 1 [file aging-14-204334-s001.pdf]

SUPPLEMENTARY TABLE

Supplementary Table 1. The primer sequences.

Gene	Primer (5′–3′)
MYH11	F: AGGCGAACCTAGACAAGAATAAG<br>R: CTGGATGTTGAGAGTGGAGATG
SM22α	F: TTCTGCCTCAACATGGCCAAC3<br>R: CACCTTCACTGG CTTGGATC
Calponin	F: ATGTCCTCTGCTCACTTCAAC<br>R: CACGTTCACCTTGTTTCCTTTC
SMMHC	F: AGGCGAACCTAGACAAGAATAAG<br>R: CTGGATGTTGAGAGTGGAGATG
Collagen I	F: TGACGAGACCAAGAACTGCC<br>R: GCACCATCATTTCCACGAGC
Vimentin	F: TCGTTTCGAGGTTTTCGCGTTAGAGAC<br>R: GACTAAAACTCGACCGACTCGCGA
Osteopontin	F: CAGCCTTCTCAGCCAAACG<br>R: CAAATCACTGCAATTCTCATGGTAGT
MiR-22	F: AAGCTGCCAGTTGAAGAACTGT<br>R: Universal PCR Reverse Primer<br>(cat. no. B532451; Sangon Biotech Co., Ltd.)
miR-23b	F: ATCACATTGCCAGGGATTACCAC<br>R: Universal PCR Reverse Primer<br>(cat. no. B532451; Sangon Biotech Co., Ltd.)
miR-125b	F: TCCCTGAGACCCTAACTTGTGA<br>R: Universal PCR Reverse Primer<br>(cat. no. B532451; Sangon Biotech Co., Ltd.)
NET1	F: AAATCCCCGGATGACGGC<br>R: TGCACTGCTCCTTCTCTTGG
SIRT1	F: GCTCGCCTTGCGGTGGACTT<br>R: GACGGCTGGAACTGTCCGGG
PTEN	F: AGCCTCTTGATGTGTGCATT<br>R: CCATTGGTAGCCAAACGGAAC
ERBB3	F: CATCGTGAGGGACCGAGATG<br>R: TGTTGCTCGAGTCCACAGTC
LAMC1	F: GAGGCAAGATATCGCCGTGA<br>R: GTATCTCGCCTGTCCACTCG
AKT3	F: GGTGCAGAGTCCCCTAGAGA<br>R: TTGGCGACAGCAGGATCATT
DPF2	F: TGCCTGTGACATTTGTGGAA<br>R: TAGCCACGATCGCAGTCATC
TGFBR1	F: TCCAACTACTGGTTTACCATTGC<br>R: ACAGCAACTTCTTCTCCCCG
TP53INP1	F: CGTCTGGGTACCTGAACGAG<br>R: ACTTCTGTGCCCGTGAGTCT
EVI1	F: GCAGACATTGCGCCTGGGGAA<br>R: CTCACAGCGGTGCTGCCGTT
GAPDH	F: AACTTTGGCATTGTGGAAGG

	R: ATTGGGGGTAGGAACA
U6	F: TGAGAACTGAATTCCATGGGTT<br>R: ACGCTTCACGAATTTGCGT
